# Micro-Drama narrative engagement and marriage anxiety among Chinese college and university students: Cross-sectional associations with romantic reality comparison and mate-selection materialism

**DOI:** 10.3389/fpsyg.2026.1929861

**Published:** 2026-10-28

**Authors:** Yanli Hua, Yihuan Yuan, Shengxuan Li, Dongming Li

**Affiliations:** 1 School of Languages and Culture, Hebei GEO University, Shijiazhuang, China; 2 College of Life Sciences, Hebei Normal University, Shijiazhuang, China

**Keywords:** digital micro-dramas, emerging adulthood, marriage anxiety, mate-selection materialism, media literacy, narrative engagement, romantic reality comparison

## Abstract

Digital micro-dramas are a prominent short-form narrative format in China, but the relationship between engagement with these narratives and young adults’ relationship beliefs is not well understood. This cross-sectional survey examined associations among general micro-drama narrative engagement (MVE), romantic reality comparison (RRC), mate-selection materialism (MSM), and future-oriented marriage anxiety (MA) in 2,015 Chinese college and university students. Media literacy (ML) was examined as an additional correlate and, exploratorily, as a moderator. Confirmatory factor analysis, structural equation modeling, and bootstrap analysis were applied. MVE was positively associated with RRC (β = 0.614), RRC with MSM (β = 0.358) and MA (β = 0.281), and MSM with MA (β = 0.140; all *p* < 0.001). The modeled sequential indirect association was small (B = 0.034, 95% percentile CI [0.017, 0.051]; standardized estimate = 0.031). ML was negatively associated with RRC, MSM, and MA, whereas MVE × ML interactions did not show evidence of moderation. The measures concerned self-reported engagement and beliefs rather than objectively observed viewing, specific platforms, or algorithmic exposure. The cross-sectional estimates do not establish temporal ordering or causal mediation. Findings point to the value of examining how mediated romance is used as a comparison standard, while requiring independent replication of the study-specific measures.

## 1 Introduction

Emerging adulthood involves exploration of identity, intimacy, and possible future family roles (Arnett, 2000). Marriage-related beliefs can therefore be relevant before a person faces an immediate decision to marry. Willoughby et al. (2015) describe these beliefs as part of a broader marital paradigm. Research among Chinese college students links marriage intentions to attitudes, perceived norms, and perceived behavioral control (Xie and Hong, 2022). More recent studies examine variation in marital attitudes in relation to family conflict (Xu and He, 2026) and describe gendered expectations and economic concerns in students’ views of marriage and childbearing (Yue et al., 2026). These studies establish the relevance of future marriage as a topic of appraisal; they do not establish the prevalence or clinical severity of marriage anxiety.

In this study, marriage anxiety (MA) denotes self-reported concern about possible loss of autonomy, economic pressure, unequal family responsibilities, and a gap between idealized love and anticipated marriage. The term describes the content of four survey items and does not denote a diagnosis, an imminent wedding decision, or a validated threshold for clinically significant anxiety. Earlier research on premarital anxieties provides historical context for studying marriage-specific concerns (Zimmer, 1986). Our question is whether these anticipatory concerns covary with media engagement and relationship beliefs, not how many Chinese students have a clinical anxiety condition.

These relationship expectations are increasingly formed within a digital media environment. One rapidly developing format in China is the digital micro-drama, a serialized audiovisual narrative designed primarily for mobile and online viewing and characterized by brief episodes, compressed storytelling, rapid plot progression, and emotionally concentrated narrative structures. Recent empirical research describes digital micro-dramas as short, fast-paced, tightly structured productions, often organized into episodes lasting only a few minutes (Li et al., 2025). Micro-dramas should therefore be distinguished from conventional television dramas and longer web series by their substantially compressed episodic form, and from ordinary short-video posts by their continuing fictional narratives, recurring characters, and serial dramatic structure. Research on short-video environments more broadly has also emphasized that psychological responses depend not simply on exposure time but on interactions among content, user characteristics, cognitive-emotional responses, and platform context (Xiong et al., 2024).

Romantic micro-dramas are defined here as micro-dramas in which courtship, partner selection, commitment, relationship conflict, or marriage is central to the narrative. Such stories can supply examples of partner attributes and relationship expectations. This romantic subset motivates the comparison construct, but the MVE items asked about micro-dramas generally and did not identify the genres actually watched. The study therefore examines general micro-drama narrative engagement alongside romantic comparison, not verified exposure to romantic micro-dramas. Broader reviews of social media and close relationships (Taylor et al., 2022) and adolescent mental health (Valkenburg et al., 2022) provide context for heterogeneous media associations; neither is direct evidence about micro-drama effects on marriage anxiety.

Social comparison theory provides the main account for the proposed model: people evaluate themselves and their circumstances against available standards (Festinger, 1954). Romantic portrayals may offer such standards, and studies of close relationships show that the interpretation of comparisons matters for relationship evaluations (Broemer and Diehl, 2003; Morry and Sucharyna, 2019). Cultivation theory supplies a broader rationale for why recurring portrayals may be relevant to social-reality beliefs (Gerbner et al., 2002). However, we did not measure cumulative exposure or recurring content, so this study is not a direct test of cultivation. Together, these perspectives motivate a proposed association sequence from narrative engagement to romantic comparison and partner-evaluation beliefs; its direction remains a theoretical assumption.

Micro-drama narrative engagement (MVE) refers to immersion, identification with characters, emotional involvement, continued viewing interest, and post-viewing cognitive engagement. It describes viewers’ psychological involvement in a narrative rather than viewing frequency. This emphasis on cognitive and emotional responses draws on the broader differential susceptibility to media effects model (Valkenburg and Peter, 2013).

The second construct, romantic reality comparison (RRC), refers specifically to the tendency to use romantic relationships, partners, or standards portrayed in micro-dramas as reference points for evaluating real or anticipated romantic relationships. Thus, MVE concerns engagement with the media narrative, whereas RRC concerns the comparison of mediated romance with non-mediated relationship reality. Social comparison theory provides the principal basis for this distinction. When a media narrative is emotionally engaging and cognitively accessible, its portrayals may become more readily available as comparison standards. Research on relationship comparison has shown that external standards and interpretations of comparative information are related to relationship evaluations, satisfaction, and commitment (Broemer and Diehl, 2003; Morry and Sucharyna, 2019). Accordingly, individuals who report stronger engagement with micro-dramas may also be more likely to use portrayed relationships as standards against which everyday or anticipated romance is judged.

*H1*. Micro-drama narrative engagement is positively associated with romantic reality comparison.

Mate-selection materialism (MSM) is used here as a study-specific label for endorsement of financial capacity, family resources, material security, and consumption as partner-evaluation criteria. General materialism concerns the value assigned to material possessions (Belk, 1985; Richins and Dawson, 1992); MSM restricts attention to partnership. However, the present items do not measure the priority of money relative to affection, trust, or other nonmaterial attributes. They also cannot cleanly distinguish materialistic values from a reasonable desire for financial security. The score is therefore interpreted as endorsement of materially oriented partner criteria, without treating economic concern itself as pathological.

This distinction is especially important because money and financial evaluation have meaningful psychological implications within romantic relationships. Materialistic value orientations are associated with poorer well-being at the general level (Dittmar et al., 2014), while more recent relationship research shows that making personal worth contingent on financial success is associated with financial conflict and poorer romantic relationship outcomes (Ward et al., 2021). Chinese research also suggests that material considerations are salient within young adults’ thinking about marriage: Xie and Hong (2022), for example, identified the materialization of marriage among beliefs relevant to Chinese college students’ marriage intentions.

The proposed ordering places RRC before MSM because comparison with idealized romantic portrayals may make wealth, family resources, consumption, and status more salient in partner evaluation. This ordering is theoretical: pre-existing partner preferences may also shape media selection and interpretation, and the cross-sectional design cannot distinguish these possibilities.

*H2*. Romantic reality comparison is positively associated with mate-selection materialism.

RRC may also be associated more directly with marriage anxiety. Romantic media can provide highly selective standards against which ordinary relationships are evaluated. When real or anticipated relationships appear less emotionally intense, less ideal, or less secure than salient fictional representations, the perceived discrepancy between romantic ideals and anticipated marital reality may become larger. Earlier research on romantic film and television has linked media exposure with idealized beliefs about love and marriage, although such associations vary by genre, content, and perceived realism (Segrin and Nabi, 2002; Hefner and Wilson, 2013; Galloway et al., 2015; Kretz, 2019). More broadly, contemporary reviews emphasize that digital media can be associated with both connection and disconnection in close relationships and that psychological processes, rather than medium exposure alone, are central to explaining these outcomes (Taylor et al., 2022).

*H3*. Romantic reality comparison is positively associated with marriage anxiety.

Stronger endorsement of material partner criteria may be associated with marriage anxiety if desired resources appear difficult to attain. Economic and material considerations are relevant to Chinese students’ marriage intentions and attitudes (Xie and Hong, 2022; Yue et al., 2026). However, our survey did not measure a discrepancy between desired and available resources, and its financial-security items need not imply unusually demanding expectations. H4 concerns an association between the measured constructs; resource shortfall is a possible explanation requiring separate measurement.

*H4*. Mate-selection materialism is positively associated with marriage anxiety.

Combining these arguments, the study proposes an indirect sequence in which MVE is associated with RRC, RRC is associated with stronger materially oriented partner-evaluation beliefs, and both are associated with marriage anxiety. Importantly, this is a statistical and theoretically specified sequence rather than evidence that the variables necessarily unfold in this temporal order.

*H5*. The modeled sequential indirect association between micro-drama narrative engagement and marriage anxiety through romantic reality comparison and mate-selection materialism is positive.

Finally, the study considers media literacy (ML) as an interpretive resource. Media literacy broadly involves the capacity to access, analyze, evaluate, and critically interpret mediated messages rather than treating them as transparent representations of reality (Livingstone, 2004; Hobbs, 2010). Contemporary digital environments also require some understanding of how automated curation and recommendation systems shape what users encounter. Dogruel et al. (2022), for example, conceptualized algorithm literacy in terms of awareness of algorithmic use and knowledge of how algorithms operate, and developed a validated measure of these competencies. Media-literacy interventions have also been shown to improve knowledge and critical evaluation of media messages (Jeong et al., 2012).

On this basis, students with higher ML may be less inclined, on average, to treat dramatized romantic portrayals, commercialized narratives, or consumption-centered partner representations as realistic standards. This expectation concerns a general negative association between ML and RRC, MSM, and MA. A stronger claim—that ML changes the strength of the MVE–MA association—requires evidence of statistical moderation and should not be assumed in advance. We therefore distinguish the hypothesized main associations of ML from an exploratory moderation question. This is a study-specific expectation, not an established consensus about romantic micro-dramas. The current ML score measures self-reported awareness and critical reflection, not performance on a literacy task.

*H6a*. Media literacy is negatively associated with romantic reality comparison.

*H6b*. Media literacy is negatively associated with mate-selection materialism.

*H6c*. Media literacy is negatively associated with marriage anxiety.

*RQ1*. Does media literacy moderate the association between micro-drama narrative engagement and marriage anxiety?

This study examines associations among general micro-drama narrative engagement, romantic reality comparison, materially oriented partner preferences, and marriage anxiety among Chinese college and university students. It distinguishes narrative engagement from relationship comparison and evaluates media literacy as an additional correlate and an exploratory moderator. Figure 1 presents the focal hypotheses; Section 2.3 specifies the complete fitted model. All paths are interpreted as cross-sectional associations.

**Figure 1:**
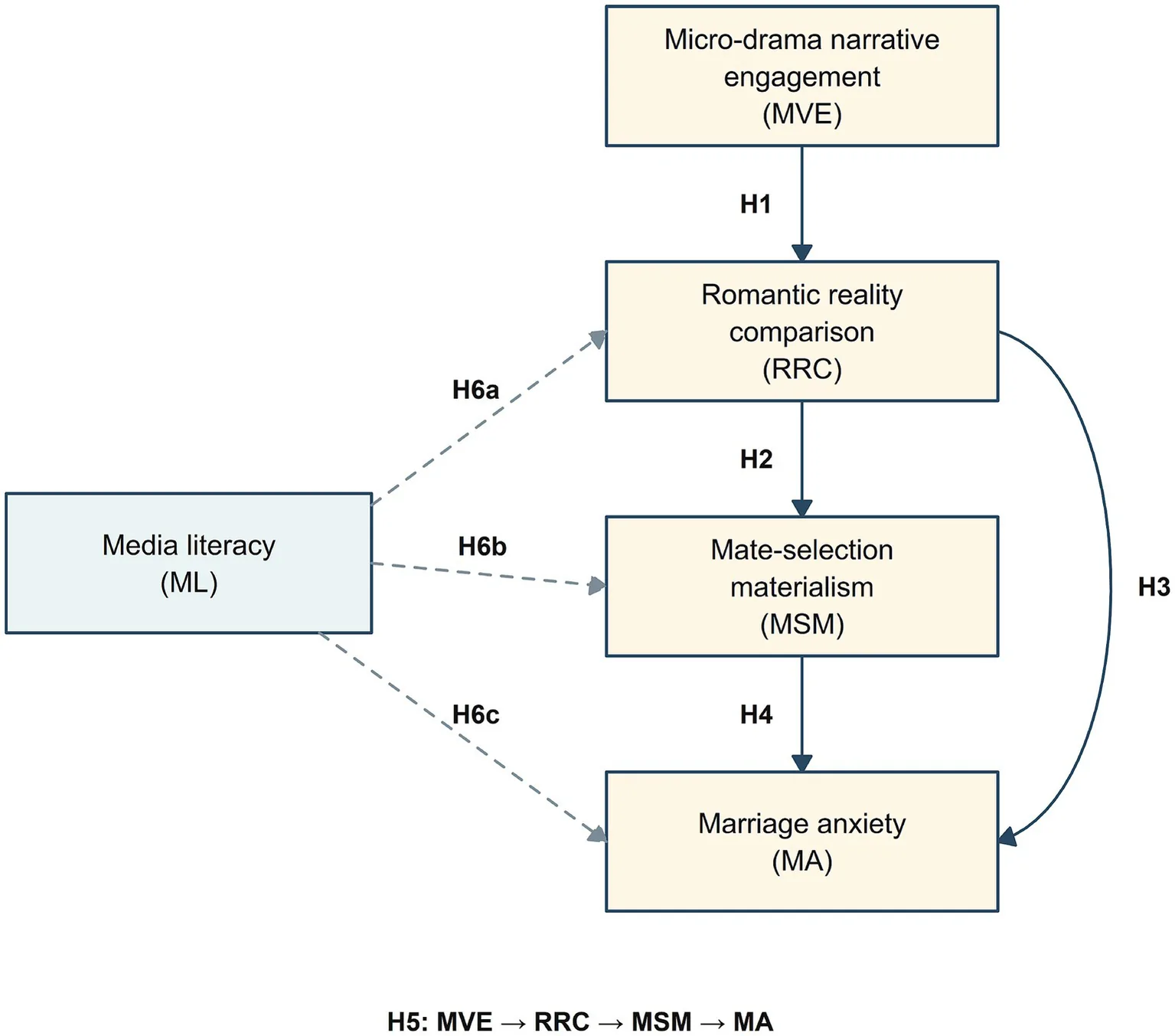
Conceptual model of the hypothesized associations. Solid arrows represent the hypothesized positive associations among *MVE*, *RRC*, *MSM*, and *MA*. Dashed arrows represent the hypothesized negative associations of media literacy with *RRC*, *MSM*, and *MA*; line styles do not indicate statistical significance or moderation. *H5* denotes the sequential indirect association along *MVE* → *RRC* → *MSM* → *MA*. Arrows indicate model specification, not established temporal or causal direction. The fitted *SEM* additionally includes *MVE* → *MSM* and *MVE* → *MA* paths and a freely estimated *MVE*–*ML* covariance, which are omitted here for clarity. The *MVE* × *ML* interactions were examined separately using composite-score regressions.

## 2 Materials and methods

### 2.1 Participants and procedure

This cross-sectional online questionnaire was administered through Wenjuanxing in January 2026. Participants were students in Chinese higher-education institutions, including undergraduate, graduate, and vocational-college categories. Convenience and snowball recruitment used university-affiliated social-media groups, student organizations, and peer networks.

Participation was entirely voluntary and anonymous. Before beginning the questionnaire, respondents were provided with an informed consent statement detailing the study’s purpose, the anonymity of their responses, and their right to withdraw at any time without penalty. To reduce potential common method bias, the questionnaire used neutral wording, emphasized anonymity, and separated conceptually distinct constructs into different sections.

A total of 2,031 questionnaires were initially collected. To ensure data quality, data cleaning procedures were applied. Responses were excluded if the participant was younger than 18 years old (*n* = 1) or if the completion time was unrealistically short, specifically less than 120 s (*n* = 15). Because all survey items were set as mandatory, there were no missing data. After screening, a final analytic sample of 2,015 valid responses was retained.

The School of Languages and Culture, Hebei GEO University approved the broader anonymous questionnaire research protocol on 22 April 2025. The approval document is titled “Development and Validation of the Sustainable Intimate Relationship Beliefs Scale” and describes research on university students’ intimate-relationship beliefs, digital-media exposure, romantic scripts, and media literacy. The questionnaire introduction described the study, anonymity, voluntary participation, and the right to withdraw. The focal analyses reported here use the cross-sectional survey responses. The questionnaire also included embedded written vignettes that are not analyzed in this article.

The analysis includes 2,015 records with complete responses to the 26 focal items. Table 1 describes the analytic sample. Categories explicitly indicating past or current experience contain 1,553 respondents; 417 reported never having dated and 45 selected “other.” The latter category was not recoded as experienced. The single gender response coded 4 represents “prefer not to disclose.” No relationship-experience or gender multi-group result is used to support the conclusions.

**Table 1:** Sample characteristics (*N* = 2,015).

Characteristic	Category	n	%
Gender	Male	860	42.68
	Female	1,154	57.27
	Prefer not to disclose	1	0.05
Age	18–20 years	740	36.72
	21–23 years	841	41.74
	24–26 years	434	21.54
Academic year	First year	367	18.21
	Second year	606	30.07
	Third year	319	15.83
	Fourth year	599	29.73
	Graduate	124	6.15
Major	Humanities/history/philosophy	357	17.72
	Education	228	11.32
	Economics/business/law	681	33.80
	Science/engineering	272	13.50
	Medicine	159	7.89
	Arts/sports	219	10.87
	Other	99	4.91
Institution type	Double First-Class/key undergraduate	438	21.74
	Ordinary public undergraduate	831	41.24
	Independent/private undergraduate	313	15.53
	Public vocational college	317	15.73
	Private vocational college	116	5.76
Family residence	Municipality/provincial capital	346	17.17
	Prefecture-level city	559	27.74
	County/county-level city	623	30.92
	Township/rural area	487	24.17
Monthly living expenses	< RMB 1,000	1	0.05
	RMB 1,000–1,500	352	17.47
	RMB 1,501–2,000	854	42.38
	RMB 2,001–3,000	767	38.06
	> RMB 3,000	41	2.03
Relationship status	Never dated	417	20.69
	Previously dated/currently single	603	29.93
	Currently dating	731	36.28
	Stable long-term relationship	219	10.87
	Other	45	2.23
Parents’ marital status	Stable marriage	1,452	72.06
	Divorced/separated	322	15.98
	One parent deceased	101	5.01
	Other/prefer not to disclose	140	6.95

Percentages may not sum to exactly 100 because of rounding. No missing values occurred for these variables in the final analytic sample.

The questionnaire did not collect participants’ university names or province of enrollment; therefore, the exact number of participating universities or provinces cannot be reconstructed and is not reported. Similarly, no direct measure of socioeconomic status based on household income, parental education, or parental occupation was administered. Monthly living expenses and family residence are therefore reported as descriptive indicators of participants’ economic and residential backgrounds rather than as a formal SES measure. Because recruitment relied on non-probability convenience and snowball sampling, the sample should not be interpreted as nationally representative of Chinese university students.

### 2.2 Measures

#### 2.2.1 Construct definitions and psychometric evaluation

The five focal measures are study-specific item sets rather than complete validated instruments adopted from the cited studies. Their intended conceptual domains are defined below and operationalized by the item wording reported in Supplementary Tables S1, S12.

The intended domains are narrative immersion, identification, emotional involvement, and continuing interest for MVE; comparison and evaluation of romance using mediated portrayals for RRC; endorsement of financial resources, family background, material security, and consumption criteria for MSM; anticipated concerns about autonomy, economic pressure, unequal responsibilities, and romantic discrepancy for MA; and self-reported awareness of fictional, commercial, and algorithmic features and critical reflection on relationship portrayals for ML. These definitions describe the intended content and do not imply that the item sets fully separate every domain.

Factorial evaluation was run during this study using a random split. The exploratory subsample contained 1,007 records and the validation subsample 1,008 non-overlapping records. Maximum-likelihood EFA used oblique promax rotation. Parallel analysis compared the observed correlation-matrix eigenvalues with the 95th percentiles from 500 simulated normal-data matrices of the same dimensions. It retained 5 factors. Supplementary Table S3 reports the complete pattern matrix. The proposed five-factor model was tested with CFA in the validation subsample (Supplementary Table S4), followed by full-sample CFA for item diagnostics.

#### 2.2.2 Distinction between MVE and RRC

Particular attention was given to distinguishing MVE from RRC. MVE represents psychological engagement with the mediated narrative itself, whereas RRC represents the use of mediated romantic portrayals as standards for evaluating real or anticipated relationships. Thus, a respondent may become immersed in and emotionally engaged with a micro-drama without necessarily applying its romantic standards to non-mediated relationship reality.

The factor results are consistent with distinguishing MVE and RRC. The square roots of AVE for MVE (0.784) and RRC (0.801) exceed their latent correlation (0.670). These diagnostics do not establish content validity or eliminate item overlap.

A sensitivity analysis omitted MVE6 (“I sometimes connect micro-drama plots with real life”), which overlaps with comparison content. Five-item reliability was α = 0.875. The standardized residual MVE–MA estimate changed from −0.075 (model-based ML *p* = 0.028) to −0.069 (model-based ML *p* = 0.047). These similar point estimates are not evidence of a protective effect; comparing their separate *p* values does not test a difference between them.

#### 2.2.3 Core measures

All focal items use five response categories coded 1–5. The analyses use the recorded numeric codes without further item reversal or response editing. Composite scores are item means. There are no missing focal-item values, and all 2,015 records were analyzed.

Micro-drama narrative engagement (MVE). MVE was measured with six items assessing psychological engagement with micro-drama narratives, including immersion, character identification, emotional involvement, post-viewing cognitive engagement, continued viewing interest, and perceived connections between micro-drama narratives and real life. Cronbach’s α was 0.903.

Romantic reality comparison (RRC). RRC was measured with six items assessing the tendency to compare real or anticipated romantic relationships and potential partners with portrayals in micro-dramas. Cronbach’s α was 0.914.

The administered Chinese wording of RRC4 was “短剧会让我更关注伴侣的经济条件、外貌或社会地位” (“Micro-dramas make me pay more attention to a partner’s financial circumstances, appearance, or social status”). Because the item concerns partner attributes, it has potential content overlap with MSM; this is evaluated as a limitation rather than dismissed by factor separation alone. Supplementary Table S1 provides the English translations, and Supplementary Table S12 reproduces the Chinese wording of all focal items.

Mate-selection materialism (MSM). MSM was assessed with four items measuring endorsement of financial capacity, family background, material security, and consumption-related criteria when evaluating potential romantic partners. Cronbach’s α was 0.909.

Marriage anxiety (MA). MA was assessed with four items measuring future-oriented concerns about marriage, including possible restrictions on personal freedom, economic pressure, unequal household or childcare responsibilities, and discrepancies between idealized romantic expectations and anticipated marital reality. Cronbach’s α was 0.875.

Media literacy (ML). ML was assessed with six items reflecting critical interpretation of micro-drama content, including recognition of narrative exaggeration, awareness of algorithmic recommendation, differentiation between fictional portrayals and real intimate relationships, critical reflection on gender roles, skepticism toward media-based romantic standards, and recognition of commercialization and consumerist narratives. Cronbach’s α was 0.892.

Full-sample reliability estimates were α = 0.903, 0.914, 0.909, 0.875, and 0.892 for MVE, RRC, MSM, MA, and ML, respectively; corresponding CR values ranged from 0.875 to 0.915, and AVE values ranged from 0.579 to 0.714.

Supplementary Tables S1, S12 document the wording of the 26 focal items used in the present analyses.

### 2.3 Data analysis

Analyses were run in R 4.5.3 using lavaan 0.7–2 and base R. CFA and SEM used maximum likelihood with the 1–5 items treated as continuous, without a mean structure. Listwise deletion was specified, but there were no missing focal-item values. The lavaan std.lv = TRUE setting fixed latent variances in CFA and the corresponding exogenous variances and endogenous disturbance variances in SEM to one; loadings were freely estimated. Item residual covariances were fixed to zero. The same settings were used consistently for the reported primary and sensitivity analyses.

Cronbach’s α was computed within each scale. Composite reliability and AVE were computed from the standardized full-sample CFA loadings and residual variances. Supplementary item diagnostics include unstandardized loadings with their model-based SEs, standardized loadings with delta-method SEs, item R^2^, corrected item–total correlations, and α if deleted. Discriminant-validity diagnostics use the Fornell–Larcker matrix. Fit indices are χ^2^, CFI, TLI, RMSEA with its 90% interval, and SRMR. EFA and validation-subsample CFA were conducted as described in Section 2.2.1.

The SEM specifies MVE → RRC → MSM → MA, with additional MVE → MSM, MVE → MA, and RRC → MA paths. ML predicts RRC, MSM, and MA. The MVE–ML covariance is freely estimated. Under this fully connected recursive specification, the structural part imposes no additional restrictions on the five-factor covariance matrix. Accordingly, equivalent CFA and SEM fit is expected and cannot validate the proposed directional ordering. The fitted arrows encode conditional associations rather than temporal or causal identification.

Primary-path SEs and *p* values are model-based ML quantities. Indirect associations were evaluated with 5,000 ordinary nonparametric case-bootstrap resamples. Bootstrap SEs are the empirical SDs of the estimates; 95% percentile intervals use the 2.5th and 97.5th empirical percentiles. There were 5000 usable primary-model resamples out of 5,000 (0 failed and 0 inadmissible). Intervals and unstandardized estimates are reported on the same scale, with standardized point estimates separately identified. Indirect inference is based on the percentile interval, not a *p* value computed from a different SE method. Table 2 includes both model-based and bootstrap SEs/*p* values for the residual MVE–MA path in its note to explain method sensitivity.

**Table 2:** Structural associations in the primary SEM.

Path	B	ML SE	ML p	95% percentile CI	Std. β
RRC ← MVE	0.865	0.036	< 0.001	[0.797, 0.938]	0.614
RRC ← ML	−0.315	0.029	< 0.001	[−0.372, −0.258]	−0.223
MSM ← MVE	0.324	0.038	< 0.001	[0.252, 0.402]	0.229
MSM ← RRC	0.359	0.030	< 0.001	[0.303, 0.413]	0.358
MSM ← ML	−0.431	0.031	< 0.001	[−0.500, −0.368]	−0.305
MA ← MVE	−0.083	0.038	0.028	[−0.158, −0.007]	−0.075
MA ← RRC	0.219	0.030	< 0.001	[0.161, 0.276]	0.281
MA ← MSM	0.109	0.027	< 0.001	[0.056, 0.162]	0.140
MA ← ML	−0.141	0.031	< 0.001	[−0.203, −0.079]	−0.129

MVE, general micro-drama narrative engagement; RRC, romantic reality comparison; MSM, mate-selection materialism; MA, marriage anxiety; ML, media literacy. Arrows denote model specification and conditional associations, not causal effects. The primary SEM included no demographic covariates. The primary residual MVE–MA path has model-based ML SE = 0.037571 and *p* = 0.027961; using the empirical bootstrap SE gives SE = 0.038624 and normal-reference p = 0.032523. These quantities use different standard-error methods; the main table consistently reports model-based ML p values.

No demographic variables were included as covariates in the primary SEM. ML is an additional predictor of RRC, MSM, and MA.

RQ1 was examined with composite-score regressions. Each outcome (MA, and separately RRC) was regressed on mean-centered MVE, mean-centered ML, and their product, with an intercept and HC3 heteroskedasticity-robust standard errors. RRC and MSM were not entered as intervening variables in the MA regression, and no demographic covariates were included. Thus, RQ1 concerns whether the MVE–MA association conditional on ML varies with ML; it does not test moderation of the residual SEM path or of an indirect association. Incremental R^2^ compares this regression with the corresponding model omitting only the product term.

The MVE6-omission sensitivity model retains the primary structural specification and uses 5,000 ordinary bootstrap resamples. VIFs were computed from the four composite predictors MVE, RRC, MSM, and ML.

## 3 Results

### 3.1 Sample characteristics and common method Bias

The analytic sample comprised 2,015 respondents. Detailed demographic characteristics are presented in Table 1. The sample included students from multiple academic disciplines, institution types, residential backgrounds, and levels of monthly living expenses. Given the convenience and snowball recruitment strategy, these characteristics are presented to define the analytic sample rather than to imply national representativeness. On the item “I usually watch micro-dramas,” 221 respondents (11.0%) selected “somewhat disagree,” 1,482 (73.5%) selected the neutral response, and 312 (15.5%) selected “somewhat agree” or “strongly agree.” This item measures self-description of usual viewing, not a confirmed prior-viewing eligibility criterion; the survey did not record actual viewing duration or specific platforms.

The first correlation-matrix eigenvalue accounted for 36.69% of total standardized-item variance. The single-factor CFA fit was χ^2^(299) = 15229.517, *p* < 0.001, CFI = 0.545, TLI = 0.505, RMSEA = 0.157, 90% CI [0.155, 0.160], SRMR = 0.138. The five-factor model fit was χ^2^(289) = 703.356, *p* < 0.001, CFI = 0.987, TLI = 0.986, RMSEA = 0.027, 90% CI [0.024, 0.029], SRMR = 0.025. Neither the eigenvalue percentage nor the model comparison rules out common method bias (Supplementary Table S2).

### 3.2 Measurement model

The EFA and parallel analysis retained five factors. Primary pattern coefficients ranged from 0.676 to 0.896, and the largest absolute non-primary loading was 0.103 (Supplementary Table S3). Validation-subsample CFA yielded χ^2^(289) = 550.520, *p* < 0.001, CFI = 0.984, TLI = 0.982, RMSEA = 0.030, 90% CI [0.026, 0.034], SRMR = 0.029 (Supplementary Table S4). These analyses constitute internal validation.

Full-sample CFA yielded χ^2^(289) = 703.356, *p* < 0.001, CFI = 0.987, TLI = 0.986, RMSEA = 0.027, 90% CI [0.024, 0.029], SRMR = 0.025. Standardized loadings ranged from 0.698 to 0.870. Supplementary Table S5 reports the loadings, Supplementary Table S6 reports reliability and AVE, and Supplementary Table S10 reports item diagnostics with explicitly separated unstandardized and standardized SEs.

The reported square roots of AVE exceeded the corresponding latent correlations, meeting the Fornell–Larcker diagnostic criterion (Supplementary Table S7). This factor-based separation does not establish content validity or remove the conceptual overlap between MVE6 and comparison, or between RRC4 and materially oriented partner evaluation. Independent validation remains necessary.

### 3.3 Descriptive statistics and correlations

Table 3 presents descriptive statistics and zero-order correlations. MVE was positively correlated with RRC (r = 0.616, *p* < 0.001), MSM (r = 0.500, *p* < 0.001), and MA (r = 0.197, *p* < 0.001). RRC was positively correlated with MSM (r = 0.571, *p* < 0.001) and MA (r = 0.323, *p* < 0.001), and MSM was positively correlated with MA (r = 0.299, *p* < 0.001). ML was negatively correlated with MVE (r = −0.222, *p* < 0.001), RRC (r = −0.333, *p* < 0.001), MSM (r = −0.441, *p* < 0.001), and MA (r = −0.244, *p* < 0.001).

**Table 3:** Descriptive statistics and correlations among focal variables.

Variable	Mean	SD	1	2	3	4	5
1. MVE	3.059	0.516	—				
2. RRC	3.092	0.485	0.616***	—			
3. MSM	2.899	0.541	0.500***	0.571***	—		
4. MA	2.911	0.513	0.197***	0.323***	0.299***	—	
5. ML	3.091	0.492	−0.222***	−0.333***	−0.441***	−0.244***	—

*N* = 2,015. ***p < 0.001.

### 3.4 Structural equation model

The primary SEM yielded χ^2^(289) = 703.356, *p* < 0.001, CFI = 0.987, TLI = 0.986, RMSEA = 0.027, 90% CI [0.024, 0.029], SRMR = 0.025. Its fit was numerically equivalent to the five-factor CFA fit, as expected under the explicitly specified unrestricted recursive latent structure. It is not an additional test of the temporal ordering.

MVE was positively associated with RRC (β = 0.614, *p* < 0.001), supporting H1. RRC was positively associated with MSM (β = 0.358, *p* < 0.001), supporting H2. RRC (β = 0.281, *p* < 0.001) and MSM (β = 0.140, *p* < 0.001) were both positively associated with MA, supporting H3 and H4. MVE also showed an additional positive association with MSM (β = 0.229, *p* < 0.001).

ML was negatively associated with RRC (β = −0.223, *p* < 0.001), MSM (β = −0.305, *p* < 0.001), and MA (β = −0.129, *p* < 0.001), supporting H6a–H6c. Thus, the primary structural model supported the hypothesized negative associations of ML with all three downstream constructs. The model explained approximately 49.6% of the variance in RRC, 50.0% of the variance in MSM, and 17.1% of the variance in MA.

After RRC, MSM, and ML were included, the residual MVE–MA coefficient was small and negative (β = −0.075, model-based ML *p* = 0.028). The zero-order and model-implied total associations were positive. The residual coefficient is a different conditional estimand and is not evidence that watching micro-dramas reduces anxiety. Table 2 reports all structural coefficients from the same fitted model.

### 3.5 Bootstrap indirect associations

The bootstrap percentile intervals excluded zero for the three modeled specific indirect associations. The RRC-only association was B = 0.189, bootstrap SE = 0.026, 95% CI [0.137, 0.242], standardized estimate = 0.173. The MSM-only association was B = 0.035, bootstrap SE = 0.010, 95% CI [0.018, 0.056], standardized estimate = 0.032.

The sequential RRC–MSM indirect association was B = 0.034, bootstrap SE = 0.009, 95% CI [0.017, 0.051], standardized estimate = 0.031. The total indirect association was B = 0.259, 95% CI [0.206, 0.313], standardized estimate = 0.236. The total association conditional on ML was B = 0.176, 95% CI [0.123, 0.229], standardized estimate = 0.160. Table 4 reports the specific indirect, total indirect, direct, and total estimates.

**Table 4:** Bootstrap indirect associations.

Association	B	Boot SE	CI excludes zero	95% percentile CI	Std. β
MVE → RRC → MA	0.189	0.026	Yes	[0.137, 0.242]	0.173
MVE → MSM → MA	0.035	0.010	Yes	[0.018, 0.056]	0.032
MVE → RRC → MSM → MA	0.034	0.009	Yes	[0.017, 0.051]	0.031
Total indirect	0.259	0.028	Yes	[0.206, 0.313]	0.236
Direct MVE → MA	−0.083	0.039	Yes	[−0.158, −0.007]	−0.075
Total association	0.176	0.027	Yes	[0.123, 0.229]	0.160

Estimates and percentile intervals are unstandardized; standardized point estimates appear separately. Bootstrap SEs and intervals use 5000 usable ordinary case resamples. The primary structural-path p values in Table 2 use model-based ML SEs, a different inferential quantity. Indirect inference here uses interval exclusion of zero. Cross-sectional indirect associations do not establish causal mediation or temporal ordering.

Accordingly, the results are statistically consistent with the theoretically specified sequence in which stronger MVE is associated with greater RRC, which is in turn associated with stronger MSM and greater MA. Because the data are cross-sectional, however, these indirect associations do not establish temporal ordering or causal mediation.

### 3.6 Sensitivity analysis and the residual MVE–MA Association

Excluding MVE6 yielded α = 0.875 for the five remaining MVE items. Model fit was χ^2^(265) = 628.658, *p* < 0.001, CFI = 0.988, TLI = 0.987, RMSEA = 0.026, 90% CI [0.023, 0.029], SRMR = 0.026.

The principal standardized associations remained similar after MVE6 was omitted; the complete corresponding estimates are reported in Supplementary Table S11.

The MVE6-omission model yielded a sequential indirect association of B = 0.033, 95% percentile CI [0.017, 0.051], standardized estimate = 0.030. The residual MVE–MA coefficient was β = −0.069, model-based ML *p* = 0.047. Comparing this *p* value with the primary-model *p* value of 0.028 is not a test of a coefficient difference. Neither coefficient warrants a causal anxiety-reducing interpretation. Supplementary Table S11 compares both models using the same estimation and inference conventions. For this sensitivity path, the normal-reference *p* value using its bootstrap SE is 0.056; the primary reporting convention remains model-based ML *p* values. This difference illustrates inferential-method sensitivity, not evidence of a changed underlying coefficient.

### 3.7 Exploratory moderation and additional robustness analyses

The exploratory moderation analyses did not support the proposition that ML moderated the association between MVE and either MA or RRC.

In the composite-score regression predicting MA, MVE was positively associated with MA (B = 0.149, SE_HC3 = 0.021, *p* < 0.001), whereas ML was negatively associated with MA (B = −0.219, SE_HC3 = 0.023, *p* < 0.001). The MVE × ML interaction was not statistically significant (B = −0.048, SE_HC3 = 0.041, *p* = 0.246). The main-effects model explained 8.10% of the variance in MA (R^2^ = 0.0810), and inclusion of the interaction increased explained variance only to 8.16% (R^2^ = 0.0816; ΔR^2^ = 0.0006). Thus, the interaction accounted for approximately 0.06 percentage points of additional explained variance and provided negligible incremental explanatory value.

The supplementary regression predicting RRC yielded the same substantive conclusion. MVE was positively associated with RRC (B = 0.536, SE_HC3 = 0.017, *p* < 0.001), whereas ML was negatively associated with RRC (B = −0.203, SE_HC3 = 0.017, *p* < 0.001). The MVE × ML interaction was not significant (B = −0.026, SE_HC3 = 0.031, *p* = 0.412). Explained variance increased only from R^2^ = 0.4201 to R^2^ = 0.4203 after inclusion of the interaction (ΔR^2^ = 0.0002), corresponding to approximately 0.02 percentage points of additional explained variance.

Taken together, these analyses do not support interpreting ML as a moderator. Instead, ML is more appropriately characterized in the present cross-sectional data as a negative correlate/predictor of RRC, MSM, and MA. The composite-score moderation results are reported in Supplementary Table S8.

Supplementary Table S8 reports the composite-score moderation results, Supplementary Table S9 reports VIF diagnostics, Supplementary Table S10 provides item-level diagnostics, and Supplementary Table S11 reports the MVE6-omission sensitivity analysis.

## 4 Discussion

### 4.1 Main findings and interpretation

In this sample of Chinese university students, stronger self-reported engagement with micro-drama narratives was associated with more frequent comparison of real or anticipated romance with portrayals in micro-dramas. RRC was associated with materially oriented partner-evaluation beliefs and with future-oriented marriage anxiety. The model also yielded a positive MSM–MA association. The sequential indirect standardized association was small (0.031), with an unstandardized B of 0.034 and 95% percentile CI [0.017, 0.051]. These findings are compatible with the proposed social-comparison account, but the fitted pathway is one possible representation of the observed covariance rather than a demonstrated psychological sequence. The MVE items did not refer exclusively to romantic micro-dramas; accordingly, the results cannot be described as effects of exposure to a defined romantic genre.

The factor-analytic results supported empirical separation of the two constructs, although some item-content overlap remained: MVE6 connected plots with real life, and the administered RRC4 addressed a partner’s financial circumstances, appearance, and status. Removing MVE6 left the principal RRC, MSM, and indirect paths similar, while the negative residual MVE–MA point estimate changed only slightly. Because RRC4 overlaps conceptually with MSM, further validation should examine it in independent samples and assess whether a narrower RRC scale changes the results. The total MVE–MA association was positive, so the small residual negative coefficient in the full model should not be interpreted as an anxiety-reducing relationship.

### 4.2 Media literacy as an associated resource

ML was negatively associated with RRC, MSM, and MA in the primary SEM. However, composite-score interactions between MVE and ML added only 0.06 percentage points to explained variance in MA and 0.02 percentage points in RRC, and neither reached statistical significance. Thus, these data do not support describing ML as a moderator or buffer of either tested association. The negative main associations may reflect critical media interpretation, but reverse direction and shared antecedents remain possible. The scale combined critical reflection on exaggeration, gender roles, commercialization, and algorithmic awareness into one score; future work should validate these dimensions separately before attributing the association to a particular literacy skill (Livingstone, 2004; Hobbs, 2010; Dogruel et al., 2022).

### 4.3 Relevance to emerging adult relationship beliefs

Future marriage can be a meaningful subject of concern while students are still developing expectations about partnership and family roles (Arnett, 2000; Willoughby et al., 2015). Research among Chinese university students likewise documents the relevance of economic and relational considerations to marriage intentions and attitudes (Xie and Hong, 2022; Xu and He, 2026; Yue et al., 2026). The present findings add micro-drama engagement and romantic comparison to this discussion. They concern self-reported expectations rather than clinical anxiety, actual marriage behavior, or population-level marriage trends.

### 4.4 Tentative practical implications

The findings identify topics for future evaluation in relationship and media-literacy education: appraising fictional romantic portrayals, discussing financial expectations, and considering standards of mutual respect and shared responsibility. The survey did not test educational interventions or measure platform algorithms and specific narrative content. The effectiveness of such educational approaches, and any recommendations concerning platforms or subgenres, therefore requires separate investigation.

### 4.5 Limitations and future research

First, the single-wave design cannot determine whether engagement preceded romantic comparison, whether comparison preceded materially oriented partner preferences, or whether any of these preceded marriage anxiety. Pre-existing beliefs may influence media selection and interpretation; uncontrolled confounding can also produce the observed associations. The bootstrap indirect paths are statistical decompositions of cross-sectional associations, not evidence of causal mediation (Maxwell and Cole, 2007). Longitudinal, preregistered, and experimentally informed work should test alternative orderings and measure pre-existing relationship beliefs.

Second, all primary constructs were self-reported in one questionnaire. A poor-fitting one-factor CFA does not eliminate common method variance, recall error, acquiescence, or social desirability. The questionnaire did not separately verify prior romantic micro-drama viewing, collect objective duration or platform records, or link responses to specific watched narratives. MVE measured general micro-drama narrative engagement. Genre-frequency questions provide limited self-reported context but were not used to identify the content of the narratives underlying each MVE response. Future work should record viewing histories and content systematically, and test whether associations differ across romance subgenres and audiences. Some RRC items presume viewing and RRC2 explicitly refers to opposite-sex comparison, so relevance cannot be assumed for non-viewers or all sexual orientations. MA2 and MA4 also share content with material concerns and romantic discrepancy; ML5 overlaps inversely with accepting media-based standards. The placement of embedded vignettes relative to the focal items is not documented sufficiently to rule out priming.

Third, convenience and snowball recruitment through student networks limits generalizability. The number of universities and provinces of enrollment cannot be recovered because these fields were not collected. Family residence and monthly living expenses do not constitute a full socioeconomic-status measure. Demographic variables were not covariates in the primary SEM, so observed paths may partly reflect age, gender, relationship experience, family background, or other unmeasured factors. The findings should not be generalized to Chinese university students nationally. Future studies should use broader, better documented sampling and planned covariate adjustment.

Fourth, the five focal measures were developed for this context, and evidence for their content validity remains limited. The reported conceptual definitions and split-sample factor analyses do not establish a documented pre-administration content-validation process. Internal consistency and item diagnostics provide preliminary psychometric evidence but do not substitute for independent validation or comparison with established instruments. MVE6, RRC4, MA2, and MA4 invite scrutiny for overlap with adjacent constructs. The MSM items combine financial security and consumption, so high scores may reflect pragmatic concerns as well as value orientation. Future item development should separate these domains, document expert review and cognitive interviewing, and evaluate narrower alternatives.

## 5 Conclusion

General micro-drama narrative engagement was positively associated with romantic reality comparison and materially oriented partner preferences. Its zero-order association with marriage anxiety and its model-implied total association conditional on media literacy were positive, whereas its residual association with marriage anxiety after accounting for romantic reality comparison, mate-selection materialism, and media literacy was small and negative. The modeled sequential indirect association was small. Media literacy was a negative correlate of romantic reality comparison, mate-selection materialism, and marriage anxiety; neither tested interaction provided evidence of moderation. These cross-sectional findings do not establish temporal ordering, causal mediation, or an anxiety-reducing effect of micro-drama engagement. Independent measurement validation and longitudinal or experimental research are needed.

## Data Availability

The data presented in this study are available from the corresponding authors upon reasonable request. The data are not publicly available due to privacy and ethical restrictions because the dataset contains sensitive information concerning participants’ intimate-relationship beliefs and related psychological measures.

## References

[ref1] Arnett J. J. (2000). Emerging adulthood: a theory of development from the late teens through the twenties. Am. Psychol. 55, 469–480. doi: 10.1037/0003-066X.55.5.469, 10842426

[ref2] Belk R. W. (1985). Materialism: trait aspects of living in the material world. J. Consum. Res. 12, 265–280. doi: 10.1086/208515

[ref3] Broemer P. Diehl M. (2003). What you think is what you get: comparative evaluations of close relationships. Personal. Soc. Psychol. Bull. 29, 1560–1569. doi: 10.1177/0146167203256918, 15018686

[ref4] Dittmar H. Bond R. Hurst M. Kasser T. (2014). The relationship between materialism and personal well-being: a meta-analysis. J. Pers. Soc. Psychol. 107, 879–924. doi: 10.1037/a0037409, 25347131

[ref5] Dogruel L. Masur P. Joeckel S. (2022). Development and validation of an algorithm literacy scale for internet users. Commun. Methods Meas. 16, 115–133. doi: 10.1080/19312458.2021.1968361

[ref6] Festinger L. (1954). A theory of social comparison processes. Hum. Relations 7, 117–140. doi: 10.1177/001872675400700202

[ref7] Galloway L. Engstrom E. Emmers-Sommer T. M. (2015). Does movie viewing cultivate young people’s unrealistic expectations about love and marriage? Marriage Fam. Rev. 51, 687–712. doi: 10.1080/01494929.2015.1061629

[ref8] Gerbner G. Gross L. Morgan M. Signorielli N. Shanahan J. (2002). “Growing up with television: cultivation processes,” in Media Effects: Advances in Theory and Research, eds. Bryant J. Zillmann D.. 2nd ed (Mahwah, NJ, USA: Lawrence Erlbaum Associates), 43–67.

[ref9] Hefner V. Wilson B. J. (2013). From love at first sight to soul mate: the influence of romantic ideals in popular films on young people’s beliefs about relationships. Commun. Monogr. 80, 150–175. doi: 10.1080/03637751.2013.776697

[ref10] Hobbs R. (2010). Digital and media Literacy: A plan of action. The Aspen Institute. Available online at: https://mediaeducationlab.com/sites/mediaeducationlab.com/files/Hobbs%20Digital%20and%20Media%20Literacy%20Plan%20of%20Action_0.pdf (Accessed October 19, 2026).

[ref11] Jeong S.-H. Cho H. Hwang Y. (2012). Media literacy interventions: a meta-analytic review. J. Commun. 62, 454–472. doi: 10.1111/j.1460-2466.2012.01643.x, 22736807 PMC3377317

[ref12] Kretz V. E. (2019). Television and movie viewing predict adults’ romantic ideals and relationship satisfaction. Commun. Stud. 70, 208–234. doi: 10.1080/10510974.2019.1595692

[ref13] Li W. Cheng S. Ling S. Zheng C. (2025). Impact of content features of digital micro-dramas on active aging: the serial mediation effect of emotional engagement and social connectedness. Front. Psychol. 16:1711672. doi: 10.3389/fpsyg.2025.1711672, 41280198 PMC12634578

[ref14] Livingstone S. (2004). Media literacy and the challenge of new information and communication technologies. Commun. Rev. 7, 3–14. doi: 10.1080/10714420490280152

[ref15] Maxwell S. E. Cole D. A. (2007). Bias in cross-sectional analyses of longitudinal mediation. Psychol. Methods 12, 23–44. doi: 10.1037/1082-989X.12.1.23, 17402810

[ref16] Morry M. M. Sucharyna T. A. (2019). Relationship social comparisons in dating and marital relationships: adding relationship social comparison interpretations. J. Soc. Psychol. 159, 398–416. doi: 10.1080/00224545.2018.1498826, 30058945

[ref17] Richins M. L. Dawson S. (1992). A consumer values orientation for materialism and its measurement: scale development and validation. J. Consum. Res. 19, 303–316. doi: 10.1086/209304

[ref18] Segrin C. Nabi R. L. (2002). Does television viewing cultivate unrealistic expectations about marriage? J. Commun. 52, 247–263. doi: 10.1111/j.1460-2466.2002.tb02543.x

[ref19] Taylor S. H. Zhao P. Bazarova N. N. (2022). Social media and close relationships: a puzzle of connection and disconnection. Curr. Opin. Psychol. 45:101292. doi: 10.1016/j.copsyc.2021.12.004, 35016090

[ref20] Valkenburg P. M. Meier A. Beyens I. (2022). Social media use and its impact on adolescent mental health: an umbrella review of the evidence. Curr. Opin. Psychol. 44, 58–68. doi: 10.1016/j.copsyc.2021.08.017, 34563980

[ref21] Valkenburg P. M. Peter J. (2013). The differential susceptibility to media effects model. J. Commun. 63, 221–243. doi: 10.1111/jcom.12024

[ref22] Ward D. E. Park L. E. Walsh C. M. Naragon-Gainey K. Paravati E. Whillans A. V. (2021). For the love of money: the role of financially contingent self-worth in romantic relationships. J. Soc. Pers. Relat. 38, 1303–1328. doi: 10.1177/0265407521991663

[ref23] Willoughby B. J. Hall S. S. Luczak H. P. (2015). Marital paradigms: a conceptual framework for marital attitudes, values, and beliefs. J. Fam. Issues 36, 188–211. doi: 10.1177/0192513X13487677

[ref24] Xie J. Hong X. (2022). Research on factors affecting Chinese college students’ marriage intention: applying the theory of planned behaviour. Front. Psychol. 13:868275. doi: 10.3389/fpsyg.2022.868275, 35719550 PMC9203155

[ref25] Xiong S. Chen J. Yao N. (2024). A multidimensional framework for understanding problematic use of short video platforms: the role of individual, social-environmental, and platform factors. Front. Psych. 15:1361497. doi: 10.3389/fpsyt.2024.1361497, 39301222 PMC11410597

[ref26] Xu H. He Y. (2026). Interparental conflict, not divorce, is linked to less positive marital attitudes among Chinese emerging adults. BMC Psychol. 14:437. doi: 10.1186/s40359-026-04232-y, 41736114 PMC13036988

[ref27] Yue Y.-R. Xia W.-T. Tao J. Zhang Y. Tang J. Su D. (2026). A mixed-methods survey to explore current status of college students’ views on marriage and childbearing and their influencing factors. BMC Public Health 26:666. doi: 10.1186/s12889-026-26326-3, 41593436 PMC12918220

[ref28] Zimmer T. A. (1986). Premarital anxieties. J. Soc. Pers. Relat. 3, 149–159. doi: 10.1177/0265407586032002

