# Supplementary material for: Micro-Drama narrative engagement and marriage anxiety among Chinese college and university students: Cross-sectional associations with romantic reality comparison and mate-selection materialism
Source: Front Psychol. 2026 Oct 28;17:1929861. doi: 10.3389/fpsyg.2026.1929861 (PMC13752121; doi:10.3389/fpsyg.2026.1929861)
Supplement: Supplementary file 1 [file Data_Sheet_1.PDF]

Supplementary Materials

Table S1. English wording of the 26 focal items

Construct	Item	English translation of item
MVE	MVE1	When watching micro-dramas, I often feel immersed in the story world.
MVE	MVE2	I unconsciously identify with the characters in micro-dramas.
MVE	MVE3	The emotional conflicts in micro-dramas clearly affect my emotions.
MVE	MVE4	After watching, the plot sometimes remains in my mind.
MVE	MVE5	I continue watching because I want to know what happens next.
MVE	MVE6	I sometimes connect micro-drama plots with real life.
RRC	RRC1	After watching micro-dramas, I make comparisons involving real-life romantic relationships.
RRC	RRC2	I compare real-life opposite-sex individuals with micro-drama characters.
RRC	RRC3	I feel that ordinary people's romantic relationships are not romantic enough.
RRC	RRC4	Micro-dramas make me pay more attention to a partner's financial circumstances, appearance, or social status.
RRC	RRC5	After watching high-consumption romance in micro-dramas, I reimagine what ideal romance should look like.
RRC	RRC6	Romantic relationships in micro-dramas sometimes make me feel disappointed with real-life romance.
MSM	MSM1	I hope my future partner will have strong financial capacity.
MSM	MSM2	A partner's income and family background would significantly influence my choice.
MSM	MSM3	Stable and superior material conditions are an important source of security in intimate relationships.
MSM	MSM4	I believe high-quality romantic relationships require a certain level of consumption.
MA	MA1	I worry that marriage will limit my personal freedom.
MA	MA2	I worry about the economic pressure that comes with marriage.
MA	MA3	I worry that marriage will bring unequal household or childcare responsibilities.
MA	MA4	I worry that the reality of marriage will differ greatly from ideal love.
ML	ML1	I know that micro-dramas often use exaggerated plots to attract viewers.
ML	ML2	I know that platform algorithms continuously recommend content that interests me.
ML	ML3	I can distinguish between micro-drama plots and real intimate relationships.
ML	ML4	I reflect on whether gender roles in micro-dramas are reasonable.
ML	ML5	I do not easily treat romantic patterns in micro-dramas as realistic standards.
ML	ML6	I can identify commercialization, consumerism, and emotional gratification narratives in micro-dramas.

Note. The English translations correspond to the administered Chinese focal items.

Table S2. Single-factor and five-factor diagnostics

Model	χ^2	df	CFI	TLI	RMSEA	SRMR
One factor	15229.517	299	0.545	0.505	0.157	0.138
Five factors	703.356	289	0.987	0.986	0.027	0.025

Note. The first observed correlation-matrix eigenvalue accounts for 36.69% of standardized-item variance. These diagnostics do not rule out common method bias.

Table S3. Complete EFA pattern matrix in the exploratory subsample

Item	F1 RRC	F2 MVE	F3 ML	F4 MSM	F5 MA
MVE1	0.099	0.678	−0.034	−0.009	0.008
MVE2	0.078	0.705	−0.012	0.009	−0.061
MVE3	−0.036	0.714	0.029	0.042	0.030
MVE4	0.012	0.773	−0.004	0.005	0.018
MVE5	−0.057	0.896	0.001	0.008	0.015
MVE6	0.017	0.865	0.001	−0.032	−0.019
RRC1	0.773	−0.020	−0.020	0.010	−0.035
RRC2	0.748	0.029	0.007	−0.029	0.058
RRC3	0.747	0.067	0.010	0.033	0.028
RRC4	0.822	−0.001	−0.004	−0.008	−0.017
RRC5	0.806	0.035	0.004	0.004	−0.015
RRC6	0.840	−0.022	0.013	0.006	0.003
MSM1	0.067	−0.037	0.014	0.814	−0.013
MSM2	0.017	−0.031	−0.045	0.803	0.026
MSM3	−0.038	0.069	−0.011	0.849	−0.017
MSM4	0.014	0.019	0.000	0.859	−0.001
MA1	−0.028	0.004	0.004	−0.028	0.763
MA2	0.035	−0.044	0.016	0.022	0.824
MA3	0.019	0.010	−0.019	0.019	0.786
MA4	−0.020	0.025	−0.012	−0.024	0.853
ML1	−0.004	0.036	0.776	0.103	0.040
ML2	−0.002	0.002	0.676	−0.028	−0.030
ML3	−0.019	−0.001	0.759	0.006	0.029
ML4	−0.003	0.008	0.737	−0.043	0.004
ML5	0.029	−0.015	0.821	0.003	−0.005
ML6	0.001	−0.027	0.777	−0.053	−0.045

Note. Maximum-likelihood EFA with promax rotation; n = 1,007. Random split seed = 20260930. Parallel analysis used 500 simulated normal datasets and the 95th-percentile eigenvalue criterion, retaining five factors. Primary coefficients range 0.676–0.896; the largest absolute cross-loading is 0.103.

Table S4. CFA results in the validation subsample

N	χ^2	df	CFI	TLI	RMSEA	90% CI	SRMR
1008	550.520	289	0.984	0.982	0.030	0.026–0.034	0.029

Note. The validation records do not overlap with the exploratory records. Both subsets were drawn from the same sample and do not constitute independent recruitment. Estimation is ML with items treated as continuous and std.lv = TRUE.

Table S5. Standardized full-sample CFA loadings

Construct	Item	Standardized loading	Delta-method SE
MVE	MVE1	0.742	0.0111
MVE	MVE2	0.732	0.0114
MVE	MVE3	0.714	0.0120
MVE	MVE4	0.793	0.0095
MVE	MVE5	0.858	0.0073
MVE	MVE6	0.851	0.0075
RRC	RRC1	0.746	0.0109
RRC	RRC2	0.775	0.0100
RRC	RRC3	0.813	0.0087
RRC	RRC4	0.811	0.0087
RRC	RRC5	0.825	0.0083
RRC	RRC6	0.830	0.0081
MSM	MSM1	0.816	0.0087
MSM	MSM2	0.828	0.0083
MSM	MSM3	0.865	0.0071
MSM	MSM4	0.870	0.0070
MA	MA1	0.744	0.0118
MA	MA2	0.803	0.0101
MA	MA3	0.805	0.0100
MA	MA4	0.838	0.0091
ML	ML1	0.698	0.0128
ML	ML2	0.710	0.0125
ML	ML3	0.760	0.0109
ML	ML4	0.764	0.0108
ML	ML5	0.798	0.0097
ML	ML6	0.829	0.0087

Table S6. Reliability and convergent-validity summaries

Scale	Items	α	CR	AVE	Loading range
MVE	6	0.903	0.905	0.614	0.714–0.858
RRC	6	0.914	0.915	0.641	0.746–0.830
MSM	4	0.909	0.909	0.714	0.816–0.870
MA	4	0.875	0.875	0.637	0.744–0.838
ML	6	0.892	0.892	0.579	0.698–0.829

Note. Full-sample CFA: $\chi^2(289) = 703.356$, $p < .001$, CFI = 0.987, TLI = 0.986, RMSEA = 0.027, 90% CI [0.024, 0.029], SRMR = 0.025. CR = $(\Sigma\lambda)^2 / [(\Sigma\lambda)^2 + \Sigma(1-\lambda^2)]$; AVE = mean($\lambda^2$), using standardized loadings in the simple-structure CFA. These diagnostics do not establish independent validity.

Table S7. Fornell–Larcker matrix

Scale	MVE	RRC	MSM	MA	ML
MVE	0.784	0.670	0.544	0.221	−0.248
RRC	0.670	0.801	0.626	0.367	−0.376
MSM	0.544	0.626	0.845	0.339	−0.496
MA	0.221	0.367	0.339	0.798	−0.285
ML	−0.248	−0.376	−0.496	−0.285	0.761

Note. Diagonal entries are square roots of AVE; off-diagonal entries are latent correlations. Factor separation does not remove item-content overlap.

Table S8. Composite-score moderation regressions

Outcome	Term	B	HC3 SE	p	R^2	ΔR^2
MA	mve	0.149	0.021	< .001	0.0816	0.0006
MA	ml	−0.219	0.023	< .001	0.0816	0.0006
MA	mve:ml	−0.048	0.041	.246	0.0816	0.0006
RRC	mve	0.536	0.017	< .001	0.4203	0.0002
RRC	ml	−0.203	0.017	< .001	0.4203	0.0002
RRC	mve:ml	−0.026	0.031	.412	0.4203	0.0002

Note. MVE and ML are mean-centered. HC3 sandwich SEs and two-sided t-reference p values use residual degrees of freedom. R^2 refers to the interaction model; ΔR^2 compares it with the main-effects model. No demographic covariates or mediators enter these regressions.

Table S9. VIFs among composite predictors

Predictor	VIF
MVE	1.707
RRC	1.922
MSM	1.753
ML	1.261

Note. Predictor set: MVE, RRC, MSM, and ML. VIF = $1/(1-R^2)$ from regressing each predictor on the other three.

Table S10. Item-level diagnostics with explicitly matched standard errors

Panel A. Loadings and uncertainty

Item	Unstd. loading	SE unstd.	Std. loading	SE std.	Item R^2
MVE1	0.4685	0.0124	0.7416	0.0111	0.5500
MVE2	0.4552	0.0122	0.7322	0.0114	0.5362
MVE3	0.4411	0.0123	0.7144	0.0120	0.5103
MVE4	0.5157	0.0124	0.7933	0.0095	0.6293
MVE5	0.5333	0.0113	0.8578	0.0073	0.7358
MVE6	0.5344	0.0115	0.8510	0.0075	0.7243
RRC1	0.4344	0.0113	0.7464	0.0109	0.5572
RRC2	0.4399	0.0109	0.7752	0.0100	0.6009
RRC3	0.4730	0.0109	0.8128	0.0087	0.6606
RRC4	0.4651	0.0108	0.8112	0.0087	0.6580
RRC5	0.4843	0.0109	0.8252	0.0083	0.6809
RRC6	0.4865	0.0109	0.8296	0.0081	0.6882
MSM1	0.4993	0.0115	0.8156	0.0087	0.6652
MSM2	0.4977	0.0112	0.8280	0.0083	0.6855
MSM3	0.5290	0.0111	0.8647	0.0071	0.7476
MSM4	0.5382	0.0112	0.8702	0.0070	0.7572
MA1	0.4524	0.0122	0.7437	0.0118	0.5532
MA2	0.4826	0.0117	0.8026	0.0101	0.6441
MA3	0.4799	0.0115	0.8048	0.0100	0.6478
MA4	0.5038	0.0114	0.8380	0.0091	0.7023
ML1	0.4152	0.0120	0.6977	0.0128	0.4867
ML2	0.4266	0.0121	0.7097	0.0125	0.5037
ML3	0.4747	0.0122	0.7601	0.0109	0.5778
ML4	0.4648	0.0119	0.7640	0.0108	0.5837
ML5	0.4875	0.0117	0.7979	0.0097	0.6367
ML6	0.5185	0.0118	0.8287	0.0087	0.6867

Note. Unstandardized loadings and their model-based ML SEs come from parameterEstimates(); standardized loadings and delta-method SEs come from standardizedSolution() for the same full-sample CFA. The SE columns are not interchangeable. All unstandardized loading p values are below .001.

Table S10 Item-level diagnostics with explicitly matched standard errors (continued)

Panel B. Within-scale item diagnostics

Item	Corrected item–total r	α if deleted
MVE1	0.6836	0.8930
MVE2	0.6820	0.8931
MVE3	0.6689	0.8950
MVE4	0.7496	0.8832
MVE5	0.8163	0.8732
MVE6	0.8057	0.8747
RRC1	0.7136	0.9053
RRC2	0.7381	0.9018
RRC3	0.7645	0.8981
RRC4	0.7679	0.8977
RRC5	0.7802	0.8959
RRC6	0.7884	0.8947
MSM1	0.7683	0.8903
MSM2	0.7767	0.8873
MSM3	0.8113	0.8751
MSM4	0.8157	0.8735
MA1	0.6936	0.8542
MA2	0.7311	0.8393
MA3	0.7369	0.8371
MA4	0.7615	0.8272
ML1	0.6597	0.8803
ML2	0.6619	0.8800
ML3	0.7152	0.8718
ML4	0.7135	0.8720
ML5	0.7460	0.8669
ML6	0.7655	0.8636

Note. Corrected item–total correlations exclude the focal item from the scale total. Items were scored using their recorded 1–5 response codes without additional reverse scoring.

Table S11. MVE6-omission sensitivity analysis

Panel A. Structural coefficients

Path	Primary β	Primary ML p	Omission β	Omission ML p
RRC ← MVE	0.614	< .001	0.612	< .001
RRC ← ML	−0.223	< .001	−0.224	< .001
MSM ← MVE	0.229	< .001	0.232	< .001
MSM ← RRC	0.358	< .001	0.356	< .001
MSM ← ML	−0.305	< .001	−0.305	< .001
MA ← MVE	−0.075	.028	−0.069	.047
MA ← RRC	0.281	< .001	0.277	< .001
MA ← MSM	0.140	< .001	0.139	< .001
MA ← ML	−0.129	< .001	−0.129	< .001

Panel B. Indirect and total associations

Association	Primary B [95% CI]	Omission B [95% CI]
MVE → RRC → MA	0.189 [0.137, 0.242]	0.186 [0.135, 0.240]
MVE → MSM → MA	0.035 [0.018, 0.056]	0.036 [0.018, 0.056]
MVE → RRC → MSM → MA	0.034 [0.017, 0.051]	0.033 [0.017, 0.051]
Total indirect association	0.259 [0.206, 0.313]	0.255 [0.201, 0.314]
Total association	0.176 [0.123, 0.229]	0.179 [0.128, 0.232]

Note. Omission-model fit: $\chi^2(265) = 628.658$, $p < .001$, CFI = 0.988, TLI = 0.987, RMSEA = 0.026, 90% CI [0.023, 0.029], SRMR = 0.026. Five-item MVE $\alpha = 0.875$. Both models use ML/std.lv = TRUE. Intervals use ordinary case-bootstrap percentiles, 5,000 requested resamples per model; usable primary/omission resamples = 5000/5000. Seeds = 20260932/20260933. For the omission-model residual MVE–MA path, ML $p = 0.047461$, bootstrap normal-reference $p = 0.055583$, and the percentile CI is [−0.155388, −0.000240]. These inference methods differ; different p-value categories do not establish different coefficients.

Table S12. Exact Chinese wording of the focal items

Construct	Item	Administered Chinese wording
MVE	MVE1	看微短剧时，我常常感觉自己进入了剧情世界
MVE	MVE2	我会不自觉地代入剧中的人物
MVE	MVE3	剧中的情感冲突会明显影响我的情绪
MVE	MVE4	看完后，剧情有时还会在我脑海中停留
MVE	MVE5	我会因为想知道后续剧情而继续观看
MVE	MVE6	我有时会把剧中的情节和现实生活联系起来
RRC	RRC1	看微短剧后，我会比较现实中的恋爱关系
RRC	RRC2	我会把现实中的异性和短剧角色进行比较
RRC	RRC3	我会觉得普通人的恋爱不够浪漫
RRC	RRC4	短剧会让我更关注伴侣的经济条件、外貌或社会地位
RRC	RRC5	看了短剧中的高消费恋爱后，我会重新想象理想恋爱的样子
RRC	RRC6	短剧中的恋爱关系有时会让我对现实恋爱产生落差感
MSM	MSM1	我希望未来伴侣有较强的经济实力
MSM	MSM2	伴侣的收入和家庭条件会显著影响我的选择
MSM	MSM3	稳定且优越的物质条件是亲密关系安全感的重要来源
MSM	MSM4	我认为高质量恋爱需要一定的消费水平
MA	MA1	我担心婚姻会限制个人自由
MA	MA2	我担心婚姻中的经济压力
MA	MA3	我担心婚姻会带来不平等的家务或育儿责任
MA	MA4	我担心现实婚姻与理想爱情差距太大
ML	ML1	我知道微短剧常通过夸张情节吸引观众
ML	ML2	我知道平台算法会不断推荐我感兴趣的内容
ML	ML3	我能区分短剧情节和现实亲密关系
ML	ML4	我会反思短剧中的性别角色是否合理
ML	ML5	我不会轻易把短剧中的恋爱模式当作现实标准
ML	ML6	我能识别短剧中的商业化、消费主义和爽点叙事

Note. Response options: 非常不同意、比较不同意、一般、比较同意、非常同意.
